# Rare Effects of Quinine on the Skin

**Published:** 1887-01-01

**Authors:** C. H. Wilkinson

**Affiliations:** Surgeon in charge of St. Mary’s Hospital, Galveston, Texas


### RARE EFFECTS OF QUININE ON THE SKIN.

By C. H. WILKINSON, M. D.,
Surgeon in charge of St. Mary's Hospital, Galveston, Texas. Reported to the Galveston Medical Club at its meeting August 9, 1886.

Gentlemen:—On May 17, 1886, I was called in to see Mrs. C., a young married woman, who had just passed a large clot from the uterus, which upon examination proved to be a product of conception of two months standing. As there was then free hemorrhage, and the os was patulous, in order to free the womb of its debris I ordered the following:

℞. Ferr. and quinine citrate.......................gr. xxx.
Ergotin.................................gr. iii.
Ext. nux. vom...........................gr. v.

Mx. and ft. caps. xii.
Sig.—One to be taken at 8, 12, 4, 8, daily, as needed.

Three of these capsules had been taken when I was called in again to see the case. On entering the sick room my patient exclaimed: "Doctor, did you not give me quinine in those capsules?" To which I replied, "I did." She then explained to me that quinine was a drug which invariably threw her into a fever, and covered her from head to feet with a brilliant, reddish eruption. Indeed, she demonstrated the fact in her scarlet appearance, brilliant eye, rapid pulse (115), and exalted temperature (101 deg.). She was literally covered with an eruption on the skin of an erythematous variety, which was attended with a most intense itching, and which had broken out upon her after taking the second capsule. No uterine pains had been excited by the remedy, but her flow had almost ceased. There was no evidence of any inflammation of the womb, and I was forced to attribute all her objective symptoms to the quinine. She added that several years ago a then prominent physician of this city had given her a small dose of quinine, and the same phenomena had followed, much to his and her surprise.

She asked me would I give her an iron tonic to build up her strength, and, to my suggesting "Brown's Iron Bitters" to her, she replied promptly that the medicine contained quinine and served her in this very way on a former occasion. "I cannot even take Peruvian bark," she added, "it also brings out the eruption."

In this instance the skin was covered with a roseolar discoloration, the eruption being scarlatiniform upon the body, and morbilliform upon the hands and face. A most intolerable itching of the scalp and corporal surfaces kept the patient busy all the time with her finger nails, and reminded one very forcibly of one of the most prominent subjective symptoms of urticaria.

I desisted in the further use of the capsules, and placed her upon a solution of the sulphite of soda, 30 grains to the ounce, which I directed to be applied to the face and hands with the view of destroying the dermatitis in those situations.

May 19, 11 a. m.—Patient's condition but little, if any, changed since last visit. Eruption still out, especially upon the trunk; says that the itching last night was very intense. Very little flow and no pains in the region of the uterus.

May 20, 3 p. m.—Eruption nearly all gone on face and hands where the soda lotion was used; but upon the trunk, and upon the lower limbs especially, it is very prominent in the morbilliform variety, and intensely itchy. It looks precisely like a case of declining measles. All fever has disappeared, and the uterus has almost ceased to make a "show."

May 22, 12 m.—Eruption plainly visible upon the trunk and lower limbs. Resembles very much a case of measles; is itchy and begins to desquamate in small scales.

May 25, 12 m.—All acute symptoms of dermatitis have disappeared. A very free desquamation of the cuticle is now going on, flakes of skin two by four inches being cast off as in the case of an extensive burn. Her hands showed evidence of this desquamation to a very marked degree. A large patch of denuded cutis, two by three inches, was visible upon the left hand, while patches of dead and puckered skin resembling a number of scalds were preparing for exfoliation on the right. She says: "I can brush handfuls of dead skin from my head every morning, and am kept busy peeling away dead pieces all over my body." Her lower limbs, which I examined, were similarly conditioned to the hands. The most disagreeable symptom referable to the skin complaint now remaining was the intense itching of the body, which kept her awake for hours last night, and which, so far to-day, has kept her pretty busy in trying to allay.

At this observation it was discovered that the patient's memory was somewhat impaired, and her ideas confused. Undoubtedly due also to the effects of quinine. She informed me that several times during the past twenty-four hours, bright erythematous streaks would appear upon her arms, always parallel with the axis of those limbs, and that she had on such occasions dreaded lest she would break out all over again.

Now, while these cases are extremely rare, they are by no means unknown to the profession. Out of ten thousand cases in hospital and private practice, where I have employed quinine internally, never had such a train of phenomena appeared after its use in a single instance. The ratio, therefore, might perhaps be given as one case in ten thousand of those who partake of this drug.

Stille says in his third edition : "Dr. Hinkle ascribes to cinchona the production in children under twelve years of age, of a peculiar erythematic hue of the skin, which confined itself to the face, thorax, and upper extremities, and continued from one to two hours while the patient was under its full influence. We, too, have been informed of cases in which any salt of cinchona occasioned an eruption of urticaria."

Bartholow says in his work on Therapeutics : "The action of quinia has occasionally been attended by the appearance of an eruption on the skin. Sometimes the exanthem has been in the form of an erythema, sometimes it has assumed the appearance of urticaria ; again it has seemed to be herpetic. There is in fact no constant and invariable eruption, and many of the reported cases are open to the suspicion that the appearances on the skin are merely accidental, and not causative."

There could have been no false conclusions in my case. Her erythema was undoubtedly due to the quinine she took, and not over four grains of the salt got access to the circulation before her dermatitis began. Indeed, it did not require so much of the drug. She stated to me at my first visit that "I cannot take Brown's Iron Bitters ; it contains quinine. I tried a dose of it once and it covered me all over with this same eruption." Now, Brown's Iron Bitters contain not over two grains of cinchona bark to the drachm, so that a dose of the bitters, a half ounce, would at the highest figure contain only eight grains of the bark, or about one-fifth of a grain of quinine.

The effect of the cinchona salt then seems to have been the cause of fever, and of a cutaneous eruption of great severity, which lasted eight days, and which assumed the intensity of the local manifestations of a severe case of scarlatina.

On the 27th, in reply to the question, she stated that "this is the fifth time I have been broken out by quinine. Three times by former physicians, and once when I took the bitters. I can take the tincture of iron, it does not affect me in this manner."

I have been a little prolix in the recital of this case, but it was on account of two reasons that I did so. First, to prove beyond a doubt that the eruption in this case was produced by no other cause than by the quinine ; and, secondly, to impress upon the profession of hereafter bearing the fact in mind that "dermatic cinchonism" is no myth, but a reality that should be borne in mind whenever we are called upon to introduce cinchona into a newly acquired family of patrons.

Supplementary to the foregoing report, I will add that after my experience with the case, to-wit: About one month later, this lady was unfortunate enough to sustain a sixth attack of dermatitis, from the same cause, cinchona. Having occasion to require the services of a physician, she called in the old family attendant, who was out of the city at the time I was called on to treat her for abortion.

Finding the os patulous, the doctor, a very skillful physician and excellent gentleman, prescribed nearly the identical remedy that I had given at my introductory visit, viz.: Ferr. et. quin. citrat, and ergotine, he not being cognizant of the lady's idiosyncracy. The result of this administration was a relapse of all the symptoms enumerated in the foregoing report, with the exception, or rather addition, of a much heavier exfoliation of the cuticle.

At the meeting of August 9, referred to above, *a cast of the foot*, composed of exfoliated cuticle — a veritable Cinderella slipper — the result of her latest cinchonism, was shown around to the members present, many of whom expressed surprise at the curious relationship between the phenomenon and its cause.— *Texas Courier-Record.*

